# Mass Drug Administration With Artemisinin-Piperaquine for the Elimination of Residual Foci of Malaria in São Tomé Island

**DOI:** 10.3389/fmed.2021.617195

**Published:** 2021-07-12

**Authors:** Mingqiang Li, Fei Tuo, Ruixiang Tan, Hongying Zhang, Shaoqin Zheng, Qi Wang, Qin Xu, Xinbing Yu, Fangli Lu, Zhibing Wu, Jun Huang, Herodes Sacramento Rampao, Carlos Alberto Bandeira D'almeida, Hong Yan, Jianping Song, Wenfeng Guo, Changsheng Deng

**Affiliations:** ^1^Artemisinin Research Center, Guangzhou University of Chinese Medicine, Guangzhou, China; ^2^Science and Technology Park, Guangzhou University of Chinese Medicine, Guangzhou, China; ^3^Department of Parasitology, Zhongshan School of Medicine, Sun Yat-Sen University, Guangzhou, China; ^4^The First Affiliated Hospital of Guangzhou University of Chinese Medicine, Guangzhou, China; ^5^Sino-French Hoffmann Institute, Guangzhou Medical University, Guangzhou, China; ^6^Centro Nacional de Endemias, Ministry of Health, São Tomé, São Tomé and Príncipe

**Keywords:** mass drug administration, artemisinin-piperaquine, *P. falciparum*, elimination, malaria foci

## Abstract

**Background:** Mass drug administration with artemisinin-piperaquine (AP-MDA) is being considered for elimination of residual foci of malaria in Democratic Republic of São Tomé and Principe.

**Methods:** Three monthly rounds of AP-MDA were implemented from July to October 2019. Four zones were selected. A and B were selected as a study site and a control site, respectively. C and D were located within 1.5 and 1.5 km away from the study site, respectively. Parasite prevalence, malaria incidence, and the proportion of the *Plasmodium falciparum* malaria cases were evaluated.

**Results:** After 3 monthly rounds of AP-MDA, the parasite prevalence and the gametocyte carriage rate of *P. falciparum* in zone A decreased from 28.29(‰) to 0 and 4.99(‰) to 0, respectively. Compared to zone B, the relative risk for the population with *Plasmodium falciparum* malaria in zone A was lower (RR = 0.458, 95% CI: 0.146–1.437). Malaria incidence fell from 290.49(‰) (the same period of the previous year) to 15.27(‰) (from the 29th week in 2019 to the 14th week in 2020), a decrease of 94.74% in zone A, and from 31.74 to 5.46(‰), a decline of 82.80% in zone B. Compared to the data of the same period the previous year, the cumulative number of *P. falciparum* malaria cases were lower, decreasing from 165 to 10 in zone A and from 17 to 4 in zone B. The proportion of the *P. falciparum* malaria cases on the total malaria cases of the country decreased of 90.16% in zone A and 71.34% in zone C.

**Conclusion:** AP-MDA greatly curbed malaria transmission by reducing malaria incidence in the study site and simultaneously creating a knock-on effect of malaria control within 1.5 km of the study site and within the limited time interval of 38 weeks.

## Introduction

Malaria is one of the world's three major infectious diseases. In 2015, there were 214 million malaria cases globally, the lowest in the last decade. However, in 2016–2018, the global number of malaria cases rose from 217 million to 228 million (1, 2), an increase of more than 5%, with 90% of the cases occurring in Africa.

**Figure 1:**
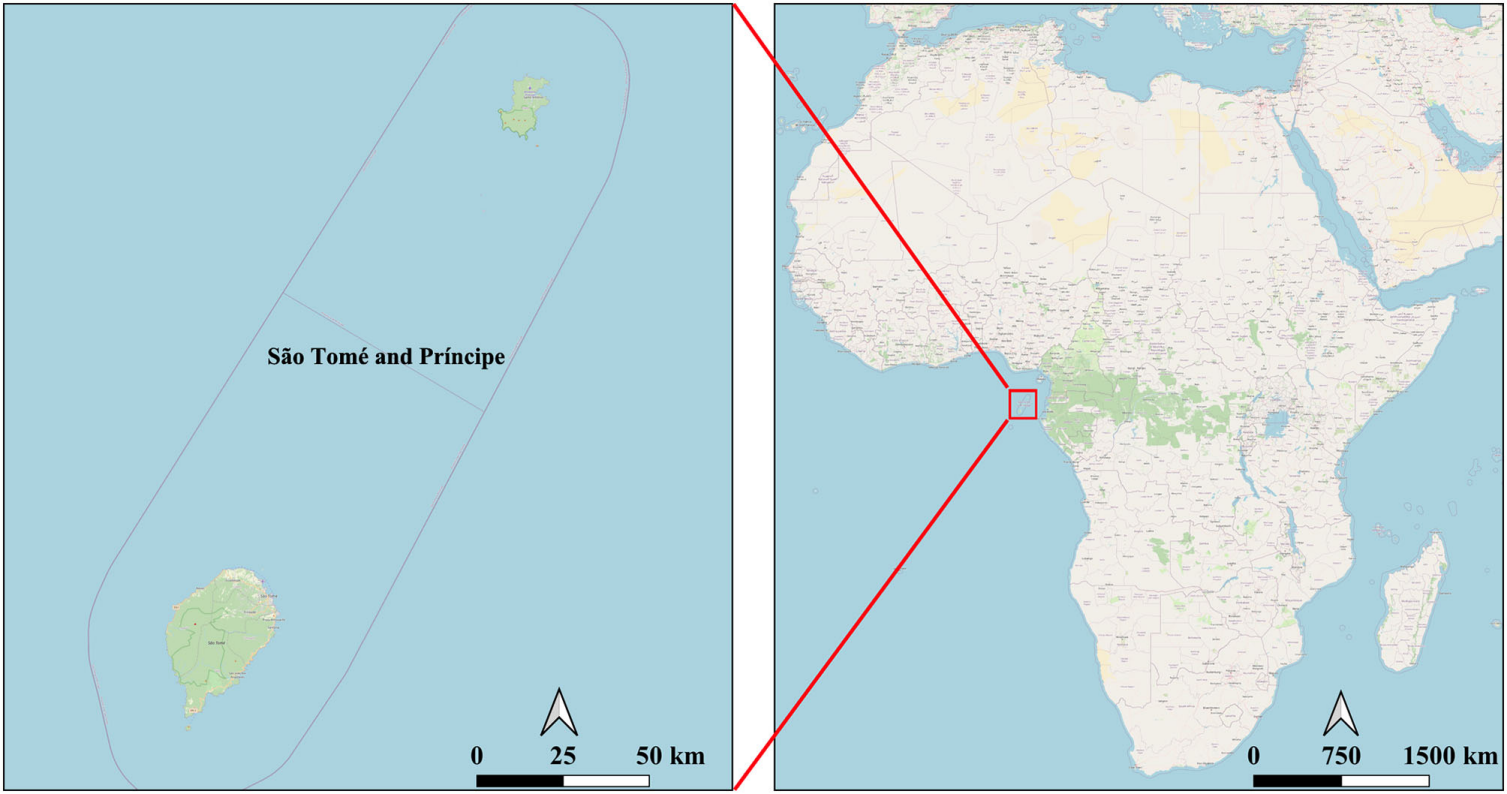
Geographical location of SãoTomé and Principe.

Situated in the Gulf of Guinea, the Democratic Republic of São Tomé and Principe (hereinafter referred to as “São Tomé and Principe”) was once a malaria endemic island country (Figure 1). Following the implementation of comprehensive measures, such as treatment with artemisinin-based combination therapies (ACTs), long-lasting insecticide-treated nets (LLINs), intermittent preventive treatment of pregnant women (IPTp), indoor residual spraying (IRS) activities and other vector control measures, malaria incidence in São Tomé and Principe gradually reduced from 37% in 2004 to 1% in 2018. Thus, the island nation was added in the list of countries with subnational/territorial elimination program by the World Health Organization (WHO) (2). The government of São Tomé and Principe has developed a PROTOCOL FOR THE CASE MANAGEMENT OF MALARIA (revised in 2018) (3) with the objective to eliminate indigenous malaria cases by 2025.

Despite the promising outlook, the situation of malaria prevention and control in 2018 was not optimistic. In 2018, the total expenditure on malaria control and treatment in São Tomé and Principe was 16 US dollars per capita (2) equaling that of 2017. However, malaria incidence in 2018 increased by 20.54% (11.57 vs. 14.56(‰)).

In 2018, Água Grande and Lemba, counties in São Tomé Island, were highly endemic, malaria incidence rates were >14.56(‰) in both counties, exceeding the national malaria incidence rate. Since the source of infection had not been eliminated in these counties, residual active foci (4, 5) remained. Malaria incidence in these residual active foci was high and increased the risk of malaria transmission to other surrounding villages, thus leading from time to time to malaria outbreaks.

To order to rapidly eliminate *P. falciparum* in residual active foci, block malaria transmission in surrounding villages, and accelerate the reduction of malaria incidence, we conducted 3 monthly rounds of mass artemisinin-piperaquine (AP-MDA) administration in Liberdade village from July to October 2019.

## Methods and Materials

### Study Sites

**Figure 2:**
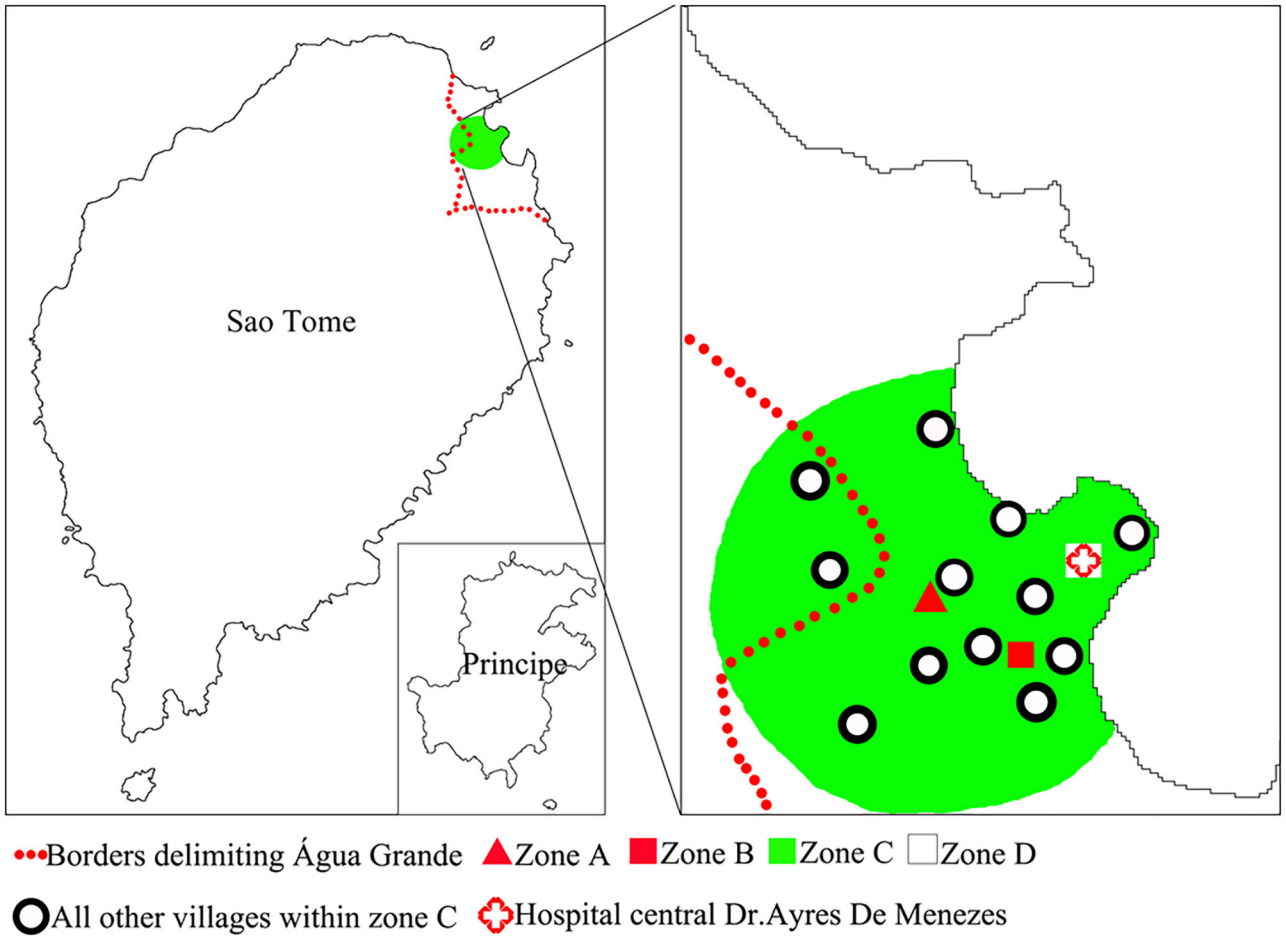
Distribution of AP-MDA village and its surroundings.

**Figure 3:**
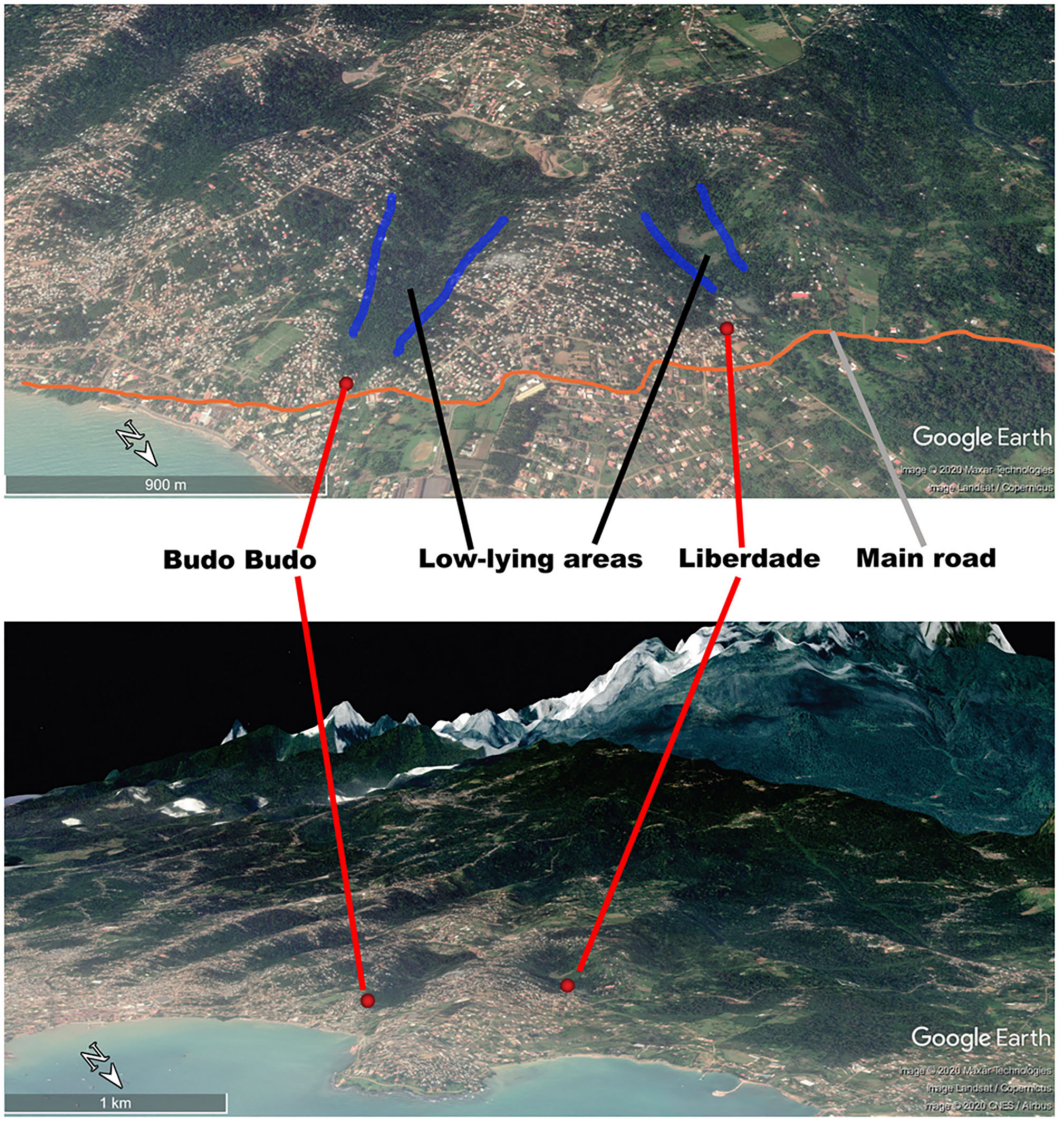
Geographical location of Liberdade and Budo Budo.

Liberdade is a village located in the north-eastern part of São Tomé Island in the central part of Água Grande County. It covers an area of 66,612 m^2^. The population of Liberdade in 2019 was 655 inhabitants. In 2014–2018, the yearly malaria incidence of Liberdade was within the range of 158.18–280.37(‰), making Liberdade the main residual focus of the area. We thus selected Liberdade village as the MDA study site (zone A) (Figure 2). Budo Budo was selected as the control village (zone B), which is situated 0.8 km south-east of Liberdade and covers an area of 26,889 m^2^. Its population in 2019 was 120 inhabitants. In 2014–2018, the yearly malaria incidence of Budo Budo was 191.30–342.59(‰). We selected the two villages mainly for their unique geographical location; both are close to each other but with no linked land or sea borders and are separated from the sea by a main road. Furthermore, both are located in low-lying areas of gully landform of volcanic island, with similar natural microenvironments. Swamps offer breeding sites for permanent water mosquitoes (Figure 3).

Zone C used to observe the extended impacts of AP-MDA was the area centered on zone A and within 1.5 km. The radius of zone C was set based on the theoretical maximum flight distance (1 km) of Anopheles mosquitoes. Zone C included 13 villages: Atras Cemiterio, Bairro Hospital, Bairro Militar, Budo Budo, Campo de Milho, Cruz Mami, Oque Del Rei, Poto Poto, Gonga, Praia Lagarto, Praia Emilia, Agua Marçal, and Ferreira do Governo. The population of zone C in 2019 was 11,732 inhabitants. In 2014–2018, the yearly malaria incidence in zone C ranged from 32.88 to 56.13(‰). Meanwhile, all areas beyond 1.5 km range were grouped into zone D (Figure 2), which represented the area completely free from AP-MDA extended impacts. The comparison of data on malaria epidemic pre- and post-AP-MDA between zone D and others can reflect the extended impacts of AP-MDA.

### Ethical Review and Informed Consent

**Figure 4:**
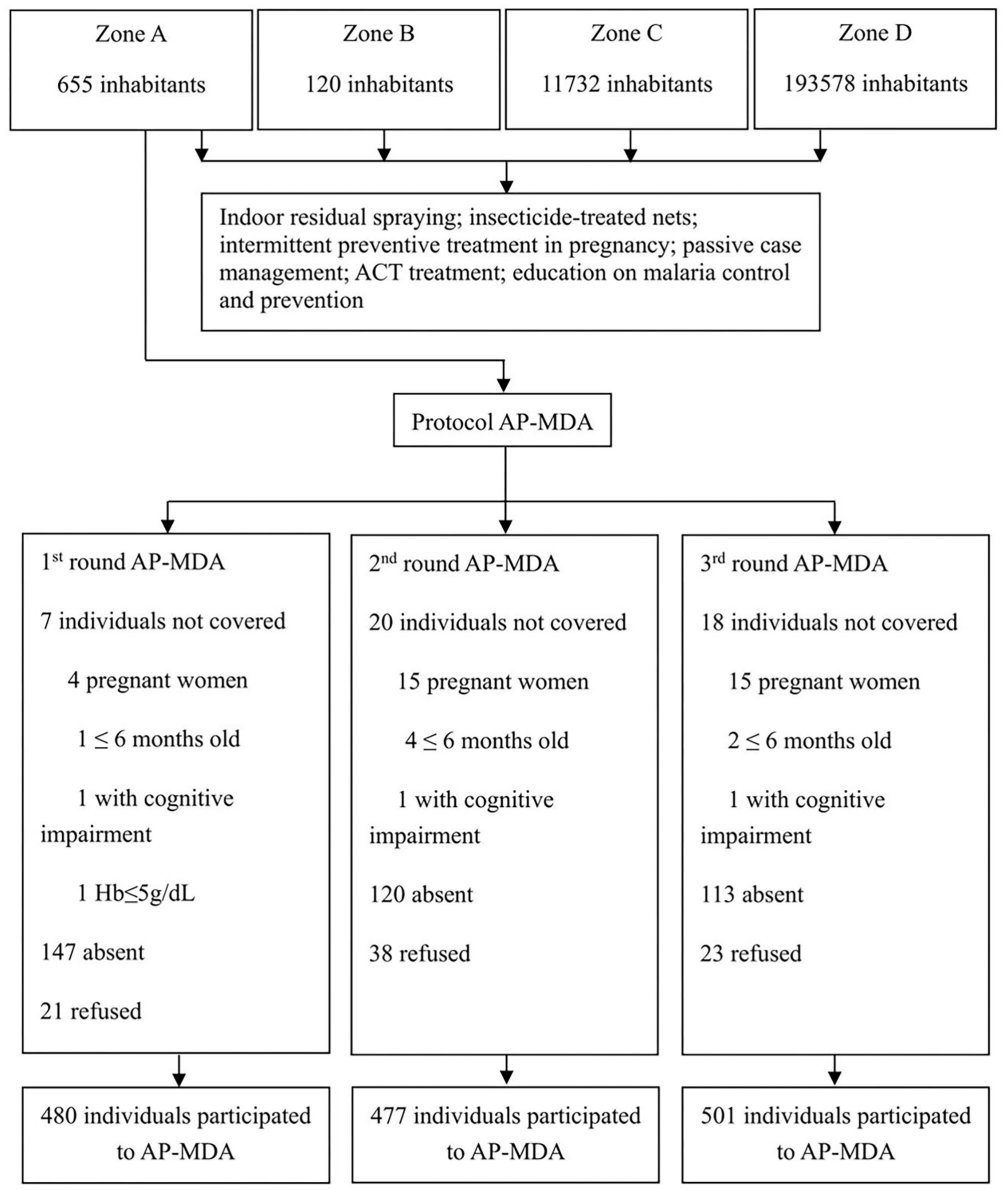
Process of study implementation.

The Ethics Committee of the Centro Nacional de Endemias (CNE) of São Tomé and Principe (Approval N°2019062001) and the Ethics Committee of the Guangdong Provincial Hospital of Chinese Medicine (Approval N°BF2019-055-01) approved the implementation of the AP-MDA project in Liberdade village. The Ministry of Health of São Tomé and Principe, the CNE, the National Malaria Control Planning Commission, the health officers of Água Grande County, and the antimalarial team from Guangzhou University of Chinese Medicine (Guangzhou, China) jointly drafted the research protocol of the present study (Figure 4). According to the protocol approved, there would be the 3 monthly rounds of AP-MDA with an interval of 28 days between each two rounds. The first round was from July 13 to August 9, 2019, the second round was from August 10 to September 6, 2019, the third round was from September 7 to October 4, 2019.

All participants in the study were required to sign informed consent forms. Informed consent form of children (<12 years old) and adolescents (12–17 years old) were signed by their guardians. Women aged 14–60 years were required to sign an informed consent form for pregnancy test. All informed consents will be kept on record for 3 years.

### Protocol of AP-MDA

#### Inclusion Criteria

Permanent residents of zone A and all temporary residents and foreign individuals in zone A during the AP-MDA;
Individuals aged more than 6 months;
Individuals that have signed informed consent.

#### Exclusion Criteria

Individuals allergic to one or more components of the drug;
Pregnant women;
Infants aged 6 months or less;
Individuals with a serious illness needing hospitalization;
Individuals with cognitive impairment.

### Drug Dosage and Treatment Regimen

Each AP tablet (manufactured by Guangdong New South Artepharm CO., LTD., Meizhou, China) is composed of 62.5 mg of artemisinin and 375 mg of piperaquine. Tablets were given orally to each participant with a strictly administered dosage based on body weight (Supplementary Table 1). Direct observation therapy (DOT) was implemented in the 3 monthly rounds of AP-MDA.

### Monitoring and Management of Adverse Drug Reactions

Community villagers were requested to report adverse drug reactions within 4 days following the drug administration. Health workers of the Hospital of Água Grande County and anti-malaria field technicians were responsible to manage and monitor the reported adverse drug reactions, and maintain data using record sheets.

### Pregnancy Test

To determine the pregnancy status of women aged 14–60 years, pregnancy tests were done before each round of AP-MDA.

### Blood Smear and Microscopy

Blood smears collected in the field were checked using microscopy in accordance with WHO's standard procedures (6).

### Other Preventive Measures

After the third round of MDA, village anti-malaria volunteers conducted daily inspection in zone A to diagnose with RDTs all feverish individuals in a timely manner. Positive individuals were treated following PROTOCOL FOR THE CASE MANAGEMENT OF MALARIA of São Tomé and Principe. All foreign individuals entering zone A (especially those staying for the night) were subjected to temperature checks and given standard malaria treatment.

### Statistical Analysis of Data

The percentage, mean and standard deviation were analyzed with SPSS 22. Counting was done using contingency tables, and the chi-square method was used for statistical analysis. Pearson's test was used when the minimum prediction frequency was higher than 5. When the minimum prediction frequency was lower than 5, Fisher's test was applied. A two-sided test *P* <0.05 was considered to indicate statistically significant differences.

## Results

### MDA Coverage

The coverage rates of the three rounds of AP-MDA in zone A were within the range of 92.62–95.81%. The proportion of individuals who refused to take the drug accounted for 0.42–0.74%. The main reason of refusal was concern about adverse drug reactions (Table 1). We further calculated the numbers of participants in each round of AP-MDA. During the first round of AP-MDA, the number of participants was 501, of which 480 individuals took the medicine. During the second round of AP-MDA, the number of participants was 515, of which 477 individuals took the medicine, among them 423 individuals took the medicine in the first round as well as in the second round. During the third round of AP-MDA, the number of participants was 524, of which 501 individuals took the medicine, among them 429 individuals took the medicine in the first round as well as in the third round, 444 in the second round as well as in the third round. A total number of 401 individuals took the medicine in all the three rounds.

**Table 1:** Artemisinin-Piperaquine-Mass Drug Administration coverage rates in zone A.

**MDA round**	**1st**	**2nd**	**3rd**
**Periods of time**	**July 13 to August 9, 2019**	**August 10 to September 6, 2019**	**September 7 to October 4, 2019**
Participants	501	515	524
Pregnant women	4	15	15
Infants ≤ 6 months	1	4	2
Individuals with cognitive impairment	1	1	1
Severe anemia (Hg ≤ 5g/dL)	1	0	0
Absent^*^	147	120	113
New arrival	0	0	0
Death	0	0	0
Participated in MDA	480	477	501
Refuse	21	38	23
MDA coverage rate	95.81% (480/501)	92.62% (477/515)	95.61% (501/524)

* *Any individual who was not home from the 1st day of drug administration to the next round of MDA was seen as absent*.

### Mobile Population Monitoring

During the three rounds of AP-MDA, 320 individuals entered zone A. Except for two individuals who refused to take drugs, all remaining participants accepted the treatment in conformity with the present study protocol (Table 2).

**Table 2:** Mobile population and compliance.

**MDA round**	**Mobile population**	**Expected number of people to take the drug**	**Number of people taking the drug**	**Refuse**	**Drug administration coverage rate**
1st	99	98^*^	98	0	100%
2nd	108	107^*^	107	0	100%
3rd	113	113	111	2	98.23%

* *One pregnant woman did not participate in MDA*.

### Drug Safety Monitoring

During the three rounds of AP-MDA, 1,458 person-times took the drug in zone A. Thirty-five person-times reported adverse drug reactions; the occurrence rate of adverse drug reactions was 2.40%. The most common adverse reactions observed were nausea, fatigue, dizziness, and headache. Adverse drug reactions were managed by local health workers. No severe adverse drug events or deaths related to MDA were reported.

### Parasite Prevalence Decreased Significantly

Before AP-MDA, 601 individuals were tested with Rapid Diagnostic Tests (RDTs), and 601 blood smear samples were collected. Nine individuals had positive RDT results and were infected with *P. falciparum* malaria. The blood smears results revealed that 14 individuals were carrying asexual *P. falciparum* and three carrying *P. falciparum* gametocytes. No other species of plasmodium and gametocytes were detected. The parasite prevalence and the gametocyte carriage rate within the population before AP-MDA were 28.29(‰) (17/601) and 4.99(‰) (3/601), respectively. However, the aforementioned individuals had no symptoms. At the end of AP-MDA in zone A, 534 individuals were subjected to RDT tests, the results of which were negative for all 534 individuals. In the meantime, blood smears results revealed no carriers of asexual *P. falciparum* as well as gametocytes. No other species of plasmodium and gametocytes were found. Parasite prevalence and gametocyte carriage rate within the population after AP-MDA were both nil.

After three rounds of AP-MDA, the active case detection for screening of parasitaemia was carried out once a month in zone A. 428 villagers were tested in November 2019, 329 villagers in December, 428 villagers in January 2020, 420 villagers in February, 422 villagers in March and 514 villagers in April. Microscopic examination was used to detect parasite. The results of each screening showed that both parasite prevalence and gametocyte carriage rate were zero. Follow-up screening was suspended due to the spread of COVID-19.

### Malaria Incidence Decreased Significantly

In zone A, the cumulative malaria cases were reduced from 165 pre-MDA (from the 29th week in 2018 to the 14th week in 2019) to just 10 cases 38 weeks post-MDA (from the 29th week in 2019 to the 14th week in 2020), all of which were *P. falciparum* malaria. Malaria incidence decreased from 290.49 to 15.27(‰) (*P* < 0.05). During the same period in zone B, the number of cases of malaria dropped from 17 to 4, all of which were of *P. falciparum* malaria. Malaria incidence decreased from 141.67 to 32.79(‰) (*P* < 0.05). Compared to zone B, the relative risk of occurrence of malaria cases in zone A 38 weeks post-MDA was lower (RR = 0.458, CI 95%: 0.146–1.437), which was not statistically significant (*P* > 0.05; Table 3).

**Table 3:** Malaria incidences ((‰)) ^*^(cumulative malaria cases/population) pre- and post-MDA.

**Area**	**3 years before MDA**	**2 years before MDA**	**1 year before MDA**	**38 weeks after MDA**
Zone A	121.82 (67/550)	103.76 (58/559)	290.49 (165/568)^**^	15.27 (10/655)
Zone B	182.61 (21/113)	25.64 (3/115)	141.67 (17/117)^**^	32.79 (4/120)^#^
Zone C	26.58 (294/11,062)	10.14 (114/11,248)	31.74 (363/11,435)^**^	5.46 (64/11,732)
Zone D	5.11 (930/182,100)	4.92 (914/185,893)	6.04 (1,147/189,781)^**^	4.94 (957/193,578)
Água Grande	10.64 (795/74,720)	6.33 (481/75,974)	12.23 (950/77,689)^**^	4.99 (395/79,226)
Whole country	6.66 (1,291/193,712)	5.49 (1,086/197,700)	8.30 (1,675/201,784)^**^	5.01 (1,031/205,965)

* *Malaria incidence 38 weeks post-MDA (from the 29th week in 2019 to the 14th week in 2020) compared with malaria incidence of the same period of each year in the past 3 years;*

** *Malaria incidence 38 weeks post-MDA was compared to malaria incidence of the same period 1 year before MDA, P < 0.05;*

# *Malaria incidence in zone B compared with malaria incidence in zone A 38 weeks post-MDA, P > 0.05*.

Based on data of malaria epidemic pre- and post- MDA, the degree of decline in malaria incidences among zones were different. The degree of decline in malaria incidence was 94.74% in zone A, 82.80% in zone C, 76.85% in zone B, 59.20% in São Tomé and Principe, 39.64% in Água Grande county, and 18.21% in zone D. All malaria cases were of *P. falciparum* infections.

### The Proportion of Malaria Cases Dropped Significantly

The proportion of cumulative malaria cases in zone A compared to the national total cases was reduced from 9.85% the same period 1 year before MDA (from the 29th week in 2018 to the 14th week in 2019) to 0.97% 38 weeks post-MDA (from the 29th week in 2019 to the 14th week in 2020) (*P* < 0.05). During the same period, the proportion of cumulative malaria cases in zone B dropped from 1.01% 1 year before MDA to 0.39% 38 weeks post-MDA (*P* > 0.05; Table 4).

**Table 4:** Proportion of cumulative malaria cases (%) ^*^(cases of each zone/ total cases) pre- and post-MDA.

**Area**	**3 years before MDA**	**2 years before MDA**	**1 years before MDA**	**38 weeks after MDA**
Zone A	5.19 (67/1,291)	5.34 (58/1,086)	9.85 (165/1,675)^**^	0.97 (10/1,031)
Zone B	1.63 (21/1,291)	0.28 (3/1,086)	1.01 (17/1,675)^#^	0.39 (4/1,031)
Zone C	22.77 (294/1,291)	10.50 (114/1,086)	21.67 (363/1,675)^**^	6.21 (64/1,031)
Zone D	72.04 (930/1,291)	84.16 (914/1,086)	68.48 (1,147/1,675)^**^	92.82 (957/1,031)
Água Grande	61.58 (795/1,291)	44.29 (481/1,086)	56.72 (950/1,675)^**^	38.31 (395/1,031)
Whole country	100 (1,291/1,291)	100 (1,086/1,086)	100 (1,675/1,675)^**^	100 (1,031/1,031)

* *Proportion of cumulative malaria cases 38 weeks post-MDA (from the 29th week in 2019 to the 14th week in 2020) compared with the proportion of cumulative malaria cases of the same period of each year in the past 3 years;*

** *Proportion of cumulative malaria cases 38 weeks post-MDA was compared to proportion of cumulative malaria cases of the same period 1 year before MDA, P <0.05;*

# *Proportion of cumulative malaria cases 38 weeks post-MDA was compared to proportion of cumulative malaria cases of the same period 1 year before MDA, P > 0.05*.

Based on data of malaria epidemic pre- and post- MDA, the degree of decline in the proportion of cumulative malaria cases were different among zones. The degree of decline in the proportion of cumulative malaria cases was 90.15% in zone A, 71.34% in zone C, 61.39% in zone B, 32.46% in Água Grande county. However, an increase of 26.22% was observed in zone D.

## Discussion

The national malaria incidences of São Tomé and Principe during 2014–2018 were 9.97–14.56(‰); all malaria cases were infected with *P. falciparum* malaria (2). According to WHO epidemiological classification of malaria (7) and based on the level of malaria incidence, São Tomé and Principe is a country of low malaria incidence and is listed as a country with subnational/territorial elimination program (2), with the aim for malaria elimination by 2025. São Tomé and Principe is in the phase of transformation from malaria control to malaria pre-elimination, with a low level of malaria transmission. However, due to the differences in the natural microenvironment and population density in different counties (8), the distribution of the endemicity of malaria is uneven in São Tomé and Principe, and the presence of malaria residual foci has led to a continued epidemic in a few localities. Countries and regions aiming to eliminate residual foci of malaria transmission can plan for a selective elimination of *P. falciparum* foci in the first stage (5). Malaria incidence in Liberdade village in 2014–2018 was in the range of 158.18–280.37(‰), which was 10–20 times higher than the national level of malaria incidence. Malaria was endemic the whole year in Liberdade. Vector breeding sites were available in the village that led to the formation of active foci (4, 5). Villages with residual transmission foci in countries with low malaria prevalence should use appropriate integrated interventions including the addition of new measures to further reduce the transmission (7). AP-MDA was never implemented in São Tomé and Principe; therefore, it was considered a new intervention in the island.

São Tomé and Principe has a seasonal climate: a heavy rain season from January to May, a dry season from June to September, and a light rainy season from October to December (9). According to the data of CNE, malaria epidemic did have seasonality in São Tomé and Principe. The malaria transmission of the whole country was in a status of lower level from the 1st week to the 13th week (January to March) and from the 28th week to the 52nd week (July to December) every year, and in a status of higher level from the 14th week to the 27th week (April to June). The number of malaria cases varies according to season; the number of cases increases in the rainy season and decreases in the dry season. As per WHO's recommendation, in an area with seasonal transmission, MDA should be implemented during the period when prevalence is the lowest (10), the reason why we conducted 3 monthly rounds of AP-MDA during the dry season (13 July 2019–5 October 2019). Each technical field team was composed of two people (6), with a total of eight field teams (seven teams responsible for entering households and administering drugs and one team responsible for the diagnosis and monitoring of the mobile population). The antimalarial drug used for MDA, artemisinin-piperaquine (AP), has a long half-life (11–13). AP has already been used in Cambodia (14), Comoros (15, 16), and Kenya (17). AP-MDA coverage rates higher than 90% were achieved mainly due to the DOT measure. The total occurrence rate of adverse drug reactions for the 3 monthly rounds of AP-MDA was 2.40%, lower than that of the MDA study conducted in Kenya (17), with no serious adverse events. The main symptoms of adverse drug reactions resolved after stopping taking the drug, indicating that AP is clinically safe.

In this study, at the end of 3 monthly rounds of AP-MDA, the parasite prevalence of *P. falciparum* malaria in zone A dropped from 28.89(‰) before MDA to zero after MDA. Additionally, the gametocyte carriage rate of *P. falciparum* dropped from 4.99(‰) to zero. Our results show that AP can kill *P. falciparum* asexual parasites and gametocytes of different stages. This conclusion is in agreement with the result of a previous AP-MDA study performed in Ngodhe, Kenya (17). AP-MDA eliminated *P. falciparum* parasites in all participants and led to a significant reduction in malaria transmission in zone A. These results confirm the high efficacy and rapid effect of AP-MDA.

After AP-MDA in zone A, we continuously recorded malaria incidence in zones B, C, D, Água Grande County, and São Tomé and Principe. Compared with the same period of the previous year, malaria incidences 38 weeks post-MDA in zone A, B, C, D, Água Grande County, and São Tomé and Principe decreased by 94.73, 77.08, 82.78, 18.03, 59.35, and 39.76%, respectively. Malaria incidence reduction in each zone reflected an overall reduction of the prevalence and intensity of malaria in São Tomé and Principe (from the 29th week in 2019 to the 14th week in 2020). Obviously, the malaria incidence reduction in zone A was the highest, indicating that compared to zone B, C, D, and Água Grande County, under the existing factors, adding 3 monthly rounds of AP-MDA, can lead to a higher decrease of malaria endemicity. Interestingly, we also found that over the same period, the percentage point reduction (82.78%) of malaria incidence in zone C was greater (*P* < 0.001) than that (18.03%) in zone D, Água Grande County (59.35%), and São Tomé and Principe (39.76%), indicating that the reduction level of malaria transmission in zone C was higher than in zone D, Água Grande County, and São Tomé and Principe. Three monthly rounds of AP-MDA in zone A not only reduced malaria incidence in zone A, but also decreased malaria incidence in zone C. Meanwhile, 38 weeks post-MDA, we observed a greater reduction of the proportion of malaria cases (94.74%) in zone A than in the other zones in the same period of the previous year. In the meantime, the proportion of malaria cases in zone C declined, and the reduction level (71.34%) was second only to that in zone A.

Monitoring drug resistance is an essential component of MDA. In our research, it was the first time that artemisinin piperaquine tablets, as MDA medicines, used in São Tomé and Principe. Artemisinin piperaquine tablets were not local first-line antimalarials, and the villagers in zone A were given the full therapeutic doses, that were in accordance with the guidelines of Mass drug administration for falciparum malaria: a practical field manual. During the three rounds of AP-MDA, we were very concerned about the condition of the villagers in zone A after taking the medicine. Anti-malarial volunteers make daily inspections in zone A, conduct RDT tests on people with fever, and collect blood films. The results of passive case detection and active case detection showed that the number of new malaria cases per week was zero in zone A from the 29th week to the 50th week in 2019. It showed that the emergence of parasitaemia did not happen in all people taking the medicine in zone A, nor did it occur in the 17 parasite carriers identified prior to MDA initiation. Furthermore, our team had accomplished 3 monthly rounds artemisinin-piperaquine mass drug administration across Anjouan Island, Union of Comoros, and PfK13 Kelch-propeller gene polymorphisms were evaluated. Analysis of 52 malaria samples after MDA showed no evidence for selection of PfK13 Kelch-propeller mutations (16). In our study, the number of new malaria cases per week was zero in zone A from the 29th week to the 50th week in 2019. However, population and mosquitoes movements inevitably brought the consequence of malaria recurrence. The first new malaria case was identified by active case detection in zone A in the 51st week in 2019. From the 51st week in 2019 to the 14th week in 2020, a total number of 10 malaria cases was accumulated. Although malaria incidences greatly reduced in zone A and in zone C, there should be more efforts indeed to maintain the status. After three rounds of AP-MDA, antimalarial volunteers were assigned to identify all fever cases in zone A, and the full therapeutic doses were given to the villagers with malaria. Zone A as a malaria focus, was the one we choose in this research, there could be more malaria foci around it. The elimination of residual active foci will be an endurable work.

Above all, our findings showed that malaria transmission was curbed after 3 monthly rounds of AP-MDA implemented in Liberdade, and AP-MDA had a knock-on effect on the surrounding areas. It did not only reduce malaria incidence in the study site but also within a specific geographical space (area within 1.5 km of the study site) and within the limited time interval of 38 weeks (from the 29th week in 2019 to the 14th week in 2020).

## Conclusion

São Tomé and Principe is a malaria endemic country, in which residual foci villages are existing in most regions of the island. In the process toward malaria elimination, the government of São Tomé and Principe shall add AP-MDA as an additional measure to other national malaria control measures to eliminate residual foci and accelerate toward malaria elimination by 2025.

## Data Availability Statement

The original contributions presented in the study are included in the article/Supplementary Material, further inquiries can be directed to the corresponding author/s.

## Ethics Statement

The studies involving human participants were reviewed and approved by the Ethics Committee of the Centro Nacional de Endemias of São Tomé and Principe and the Ethics Committee of the Guangdong Provincial Hospital of Chinese Medicine. Written informed consent to participate in this study was provided by the participants' legal guardian/next of kin.

## Author Contributions

CD, WG, JS, FT, and ML conceived and designed the experiments, SZ, RT, HY, HZ, HR, and CD'a performed the experiments, QW, ZW, QX, FL, JH, and XY analyzed the data. FT, JS, WG, and QW wrote the paper. All authors reviewed the manuscript.

## Conflict of Interest

The authors declare that the research was conducted in the absence of any commercial or financial relationships that could be construed as a potential conflict of interest.
