# Supplementary material for: Mass Drug Administration With Artemisinin-Piperaquine for the Elimination of Residual Foci of Malaria in São Tomé Island
Source: Front Med (Lausanne). 2021 Jul 12;8:617195. doi: 10.3389/fmed.2021.617195 (PMC8311023; doi:10.3389/fmed.2021.617195)
Supplement: Supplementary file 1 [file Table_1.DOCX]

Supplementary table 1. Doses of the artemisinin-piperaquine tablets.

| **Weight** | **Hour 0** | **Hour 24** |
| --- | --- | --- |
| **5–15 kg** | $\frac{1}{2}$ tablet | $\frac{1}{2}$ tablet |
| **16–30 kg** | 1 tablet | 1 tablet |
| **31–45 kg** | 1$\frac{1}{2}$ tables | 1$\frac{1}{2}$ tablets |
| **46-60 kg** | 2 tablets | 2 tablets |
| **61–75 kg** | 2$\frac{1}{2}$ tablets | 2$\frac{1}{2}$ tablets |
| **≥76 kg** | 3 tablets | 3 tablets |
